# Epilepsy Treated by the Bark of Black Elder

**Published:** 1855-01

**Authors:** 


Epilepsy treated by the Bark of Black Elder Tree.—The emeto-cathartic and hydragogue properties of the second bark of blach elder (sambucus nigra), have long been known, and used in various forms of dropsies; but no mention has hitherto been made of the employment of this substance in the treatment of epilepsy. M. Bogetti relates five examples of cure obtained by this remedy alone. In order to prepare it for administration, the branches of the elder, of one or two years, are taken; the grey bark is removed and the second bark which remains is scraped off. About five ounces of common water, hot or cold, are poured upon two ounces of the bark, and the infusion is allowed to stand forty-eight hours. The infusion, properly strained, should be taken at intervals of a quarter of an hour for a certain number of times when the fit is threatening, the patient fasting. It should be resumed every six or eight days.—*Rev. Thér.*

